# Supplementary material for: Dispersion relation of the collective excitations in a resonantly driven polariton fluid
Source: Nat Commun. 2019 Aug 27;10:3869. doi: 10.1038/s41467-019-11886-3 (PMC6712214; doi:10.1038/s41467-019-11886-3)
Supplement: Supplementary file 1 — Supplementary Information [file 41467_2019_11886_MOESM1_ESM.pdf]

Supplementary information: Dispersion relation of the collective excitations in a resonantly driven polariton fluid

Petr Stepanov,[1] Ivan Amelio,[2] Jean-Guy Rousset,[1,3] Jacqueline Bloch,[4] Aristide Lemaître,[4] Alberto Amo,[5] Anna Minguzzi,[6] Iacopo Carusotto,[2] and Maxime Richard[1]

[1] *Univ. Grenoble Alpes, CNRS, Grenoble INP, Institut Néel, 38000 Grenoble, France*

[2] *INO-CNR BEC Center and Dipartimento di Fisica, Università di Trento, 38123 Povo, Italy*

[3] *Institute of Experimental Physics, Faculty of Physics, University of Warsaw, ulica Pasteura 5, PL-02-093 Warsaw, Poland*

[4] *Centre de Nanosciences et de Nanotechnologies, CNRS, Université Paris-Sud, Université Paris-Saclay, C2N Marcoussis, F-91460 Marcoussis, France*

[5] *Univ. Lille, CNRS, Physique des Lasers Atomes et Molécules, F-59000 Lille, France*

[6] *Univ. Grenoble Alpes, CNRS, LPMMC, 38000 Grenoble, France*

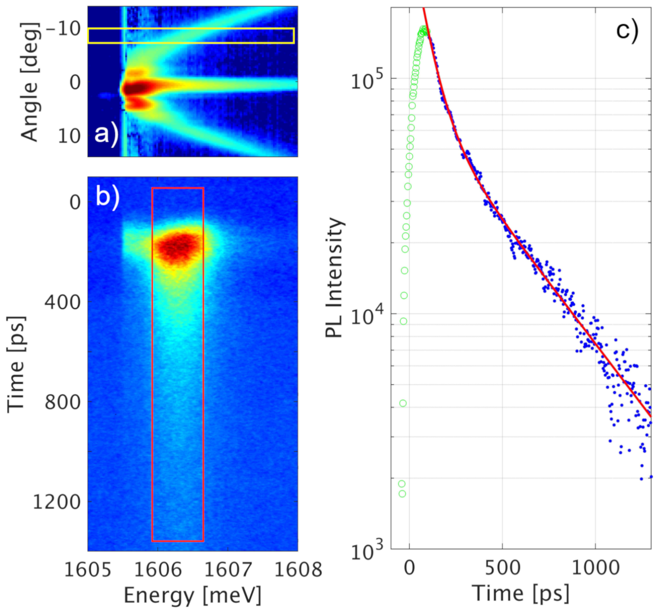

Supplementary Figure 1. **Time-resolved excitations PL** - a) Angle and energy resolved excitation photoluminescence obtained under weak picosecond-pulse excitation. The yellow rectangle show a cross-section at 7° which is sent onto the streak camera. The resulting time-resolved trace is shown in b) in a time energy color plot, and the relevant cross-section $I(t)$, integrated in energy over the polariton emission linewidth (red rectangle) is shown in c) along with a fit (red line) consisting of a sum of two exponential decays with characteristic times $\tau_1 = 58\,\text{ps}$ (instrument-limited) and $\tau_2 = 400\,\text{ps}$.

SUPPLEMENTARY NOTE 1. DECAY TIME OF RESONANTLY EXCITED POLARITONS: EVIDENCE FOR COUPLING WITH A LONG-LIVED RESERVOIR

In order to get an independent indication that under resonant optical drive of polaritons, a reservoir of long-lived excitons is also excited in the microcavity, we performed a time-resolved photoluminescence decay measurement. The excitation strategy is similar to that used to measure the free particle dispersion relation: quasi-resonant laser excitation, red detuned central frequency with respect to the polariton ground state, weak peak intensity in order to minimize nonlinear dynamics, and small angular spread. The laser consists in picosecond pulses of 0.5 meV linewidth (FWHM). We checked that the laser high-energy tail has a negligible overlap with the upper polariton branch. Supplementary Figure 1.a shows the thus obtained time-integrated excitation photoluminescence (EPL) pattern $I_e(\theta, \omega)$. We select a polariton state at an angle of 7°, where contribution from the laser light is negligible (yellow rectangle in the figure) and send it into the streak-camera. The corresponding raw time-resolved trace is shown in Supplementary Figure 1.b and analyzed in Supplementary Figure 1.c. We fit it with a sum of two exponential decays, and find two characteristic timescales $\tau_{\text{res}} = 58\,\text{ps}$ and $\tau_2 = 400\,\text{ps}$. The first one corresponds to the instrumental time-resolution, which is fixed by the (sharp) spectral resolution of the detection. It thus reflects a dynamics τ_1 much faster than τ_{res}, that can be unambiguously attributed to the polariton state decay. τ_2 is too slow to be photon or polariton related, and corresponds to the typical timescale of long-lived excitons.

In order to have a clear understanding of these two timescales, we simulate the population dynamics of the coupled polariton/reservoir system by simplifying the scalar model of eq.(3) and eq.(4) into two coupled rate equations, in which we neglect the stimulation term in the back conversion mechanism considering the weak amplitude of the pump (i.e. the pump rate is much lower that the total loss rate):

$$\partial_t n_\text{p} = -(\gamma_\text{c} + \gamma_\text{in}) n_\text{p} + \gamma_\text{bk} n_\text{R} \quad (1)$$
$$\partial_t n_\text{R} = \gamma_\text{in} n_\text{p} - (\gamma_\text{R}^\text{nr} + \gamma_\text{bk}) n_\text{R}, \quad (2)$$

where n_p is the condensate population, n_R the long-lived excitons reservoir population, $\gamma_\text{R}^\text{nr}$ is the non-radiative decay rate of reservoir excitons, γ_in is the conversion rate from polaritons to reservoir excitons, $\gamma_\text{bk} = \gamma_\text{in}\, \rho_\text{pol}/\rho_\text{R}$ is the reverse rate from reservoir excitons to polaritons, ρ_pol and ρ_R are the densities of states of polariton and reservoir states, $\gamma_\text{R} = \gamma_\text{R}^\text{nr} + \gamma_\text{bk}$, and the other parameters are consistent with those used in the main text, and with the illustration in Fig.1.a and Fig.1.b. Note that the stimulated scattering terms proportional to the product $n_\text{p} n_\text{R}$ that stem from the bosonic enhancement factors in the quantum kinetic equations cancel out and eventually disappear from the final form of Eqs. (1-2). Note that the $\gamma_\text{bk} n_\text{R}$ term in the equation for the polariton population (1) refers to the incoherent polariton population corresponding to the excitations. If needed, it could be included in the equation for the field ψ (Eq.(3) of the main text) as stochastic terms. The general solutions of this set of differential equations are of the form $A \exp(-\Gamma_1 t) + B \exp(-\Gamma_2 t)$, where

$$2\Gamma_{1,2} = \gamma_\text{c} \pm \sqrt{\gamma_\text{c}^2 + 4\left[\gamma_\text{bk}\gamma_\text{in} - (\gamma_\text{bk} + \gamma_R^\text{nr})(\gamma_\text{c} + \gamma_\text{in})\right]}. \quad (3)$$

Since the radiative decay of polaritons is the fastest timescale, the two $\Gamma_{1,2}$ can be simplified into

$$\Gamma_1 = \gamma_\text{c} \quad (4)$$
$$\Gamma_2 = \gamma_R^\text{nr} + \gamma_\text{bk} = \gamma_\text{R}. \quad (5)$$

The slow decay time component can therefore be clearly attributed to the presence of long-lived excitons from the reservoir.

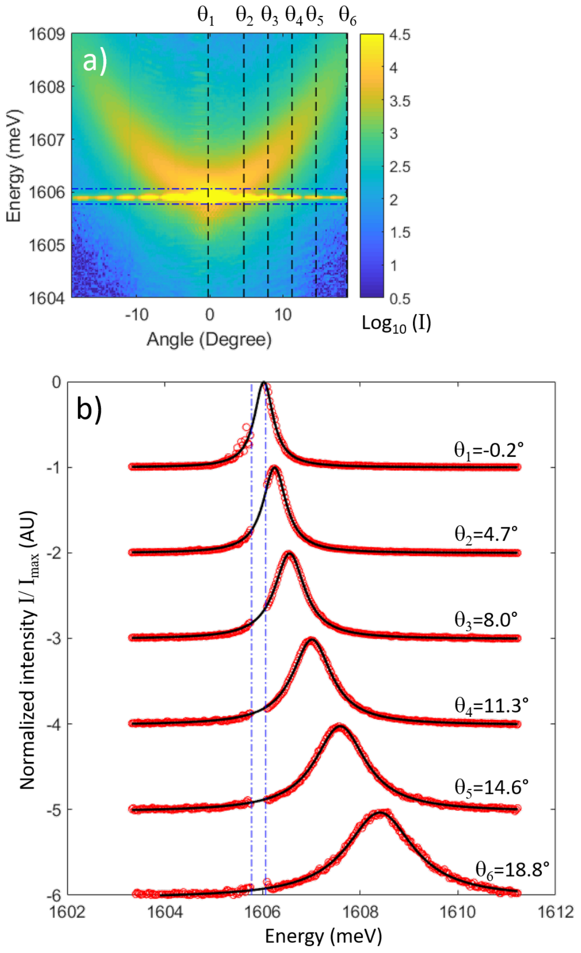

Supplementary Figure 2. **Experimental derivation of the dispersion relation** - a) Raw measurement of $\log_{10}[I_{\rm e}(\theta,\hbar\omega)]$ of WPA point '3' (color scale). b) spectra extracted from a) at angles $\theta_i = -0.2°, 4.7°, 8.0°, 11.3°, 14.6°, 18.8°$ (red symbols), and Lorentzian fits (solid black line). The dash-dotted blue lines show the laser rejection interval.

SUPPLEMENTARY NOTE 2. DETERMINATION OF THE DISPERSION RELATION AND ITS CONFIDENCE INTERVAL FROM THE MEASUREMENTS

We extract the experimental dispersion relations from the measured $I_{\rm e}(\theta,\omega)$. A careful control of the uncertainty is a critical point in this work, as the change of shape of the dispersion relation due to the interactions is a small effect, i.e. typically much smaller than the linewidth. We thus have to make sure that the dispersion shape extracted from the measurement is not the result of some noise source or of scattered laser light. We thus carry out the following analysis :

As explained in the Methods section, for each column of CCD pixels i (that contains the spectrum at a given wavevector $k_\parallel^{(i)}$), the EPL emission peak is fitted with a Lorentzian peak. from this fit, and the numerical agreement with the Lorentzian shape, we get the central frequency $\omega_i/2\pi$ of the peak as well as its 95%-confidence interval. The linewidth $\Delta\omega_i$ and its 95%-confidence interval are obtained as well from this procedure. The results for WPA and WPB are shown in the main text in Figs.3, where the line thickness of the experimental plots shows the 95% confidence interval.

Such an analysis is illustrated in Supplementary Figure 2 for the data from the working point WPA.3 defined in the main text. Supplementary Figure 2.a show the raw measurement of $I_{\rm e}(\theta,\omega)$ in color log scale. Six spectra extracted from six different angles (θ_1 to θ_6) are shown in Supplementary Figure 2.b in red. The filter rejecting the laser is shown as the interval between the blue dash-dotted lines. The Lorentzian fit is shown as a solid black line for each spectra. The quantitative results of this fit are summarized in the table below

i	θ_i [Deg]	R^2	$\hbar\omega_0$ [meV]	$\hbar\Delta\omega_0$ [meV]
1	-0.2°	1.31%	1606.027	0.011
2	4.7°	0.07%	1606.246	0.003
3	8.0°	0.09%	1606.555	0.004
4	11.3°	0.10%	1607.012	0.005
5	14.6°	0.11%	1607.591	0.007
6	18.8°	0.22%	1608.402	0.012

i	$\hbar\gamma$ [meV]	$\hbar\Delta\gamma$ [meV]
1	0.466	0.014
2	0.573	0.004
3	0.748	0.007
4	0.974	0.009
5	1.274	0.012
6	1.676	0.025

The uncertainties $\hbar\Delta\omega_0$ and $\hbar\Delta\gamma$ are the 95% confidence interval (sometime referred to as the 2σ confidence interval) of $\hbar\omega_0$, the central energy of the peak, and $\hbar\gamma$ its full-width at half-maximum, as obtained from the fitting algorithm.

SUPPLEMENTARY NOTE 3. EXCITATION MECHANISMS OF THE CONDENSATE BY THERMAL PHONONS

In this section, we present in details the arguments that support the fact that thermal phonons are the likeliest origin of spontaneously created excitations on top of the condensate observed in this work. We have shown in another work, involving microcavities made up of another materials, and at higher temperatures, that there are several possible mechanisms that can create such excitations [2]:

1. Auger scattering of two polaritons from the condensate: the recombination of a polariton excites a hot electron-hole pair, that relax into the long-lived

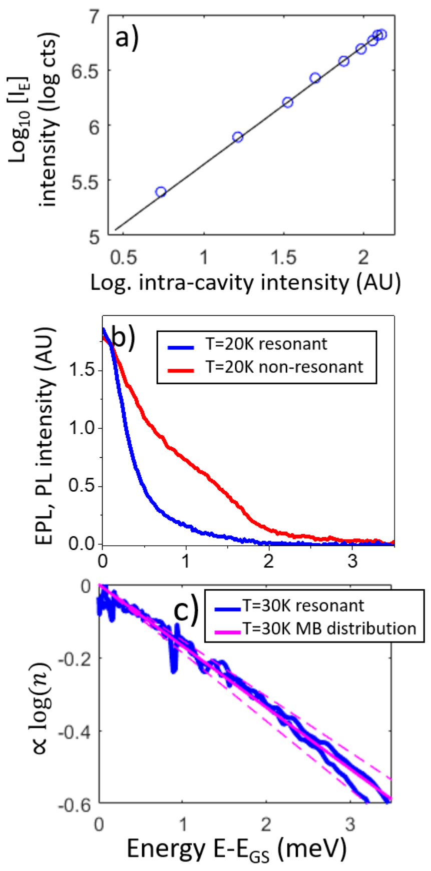

Supplementary Figure 3. **Linearity of the transfer mechanism and polaritonic population distribution** - a) Blueshift-corrected polariton excitation density versus cavity-coupled laser intensity. b) Angle-integrated spectra of polaritons photoluminescence under resonant (blue) and non-resonant laser excitation (red). c) Polariton occupancy n (within a constant factor) versus energy in the free particle regime (blue). Calculated Maxwell-Boltzmann distribution at $T = 30\,\mathrm{K}$ (solid magenta), $T = 33\,\mathrm{K}$ and $T = 27\,\mathrm{K}$ (dashed magenta).

reservoir of free excitons (at $\omega \geq \omega_{\mathrm{X}}$, and momentum beyond the light cone), and end up exciting fluctuation on top of the condensate. We see that this case involves a reservoir of free excitons that can interact with the polariton condensate as described in our work.

2. Inelastic scattering by thermal optical phonons: This mechanism takes a polariton from the condensate and create a free exciton in the reservoir described above. This inelastic scattering is possible in any microcavity as long as the polariton condensate energy is separated from the large momentum excitons reservoir by less than the energy of an optical phonons. This condition is largely met in GaAs-based microcavity. Note however that at T=30K, the temperature of our experiment, the occupancy of the optical phonons population is very small: it amounts to $\exp(-\hbar\omega_{\mathrm{LO}}/k_{\mathrm{B}}T) \simeq 1 \times 10^{-6}$, where $\hbar\omega_{\mathrm{LO}} = 36.5\,\mathrm{meV}$ is the longitudinal optical phonon energy in GaAs.

3. Inelastic scattering by thermal acoustic phonons: Such a mechanism allows out-scattering of polaritons towards any state within the frequency window $[0, \omega_{\mathrm{LO}}]$. Considering the frequency dependence of this thermal population, scattering towards states of low frequency with respect to the condensate are largely favoured. These states are (i) excitations of the polariton condensate, and (ii) localized states of excitons caused by imperfections in the quantum well, that can lie several meV below the free exciton energy, i.e. close or at resonance with the polariton condensate. These imperfections typically consist in thickness and/or alloy fluctuations [3, 4].

In scenario (1), we expect that the relation between the excitation power entering the cavity I_{in} and the polariton condensate population I_0 is sublinear. We thus checked this possibility by exciting polaritons resonantly in the redshifted configuration, i.e. $\Delta = \hbar\omega_{\mathrm{l}} - \hbar\omega^0 = -0.48\,meV$, and by measuring the angle-integrated EPL intensity versus the excitation power. What matters is the relation between the laser intensity effectively entering the cavity spacer I_{e}, that depends both on the outside excitation power I_{out} and on the blueshifting polariton mode $E_{\mathrm{LP}}(I_{\mathrm{out}})$. This relation can be accurately modeled in the weak to moderate excitation power by assuming a lorentzian lineshape of fixed linewidth for the polariton mode, such that

$$I_{\mathrm{in}} \propto \left[(E_{\mathrm{las}} - E_{\mathrm{LP}}(I_{\mathrm{out}}))^2 + (\hbar\gamma_{\mathrm{c}})^2\right]^{-1}, \qquad (6)$$

where both $E_{\mathrm{LP}}(I_{\mathrm{out}})$ and $\hbar\gamma_{\mathrm{c}}$ are direct experimental observables. Assuming that the angle-integrated EPL intensity is proportional to the condensate density, we can thus measure the relation between I_0 and I_{in}. The result is shown in Supplementary Figure 3.a: it turns out that over 1.5 orders of magnitude, this relation is linear (or slightly superlinear), with a fitted exponent of 1.07 ± 0.03. Note however that this particular argument should be taken with caution, it is based on the assumption that the magnitude of the birefringence that couples the co- and cross-polarized condensate does not depend on the nonlinearity, which might not necessarily be true.

Another, and perhaps even stronger argument which is inconsistent with scenario (1), as well as with scenario (2) is the following: both scenarios involve an intermediate reservoir of long-lived free excitons beyond the light cone. In these scenario, this reservoir relaxes into condensate excitations by two-body scattering or by emission of phonons. A way to check this possibility is to compare the condensate excitation spectrum $I_{\mathrm{R}}(\hbar\omega)$ obtained under resonant excitation, with the one measured under non-resonant excitation, when the laser is tuned

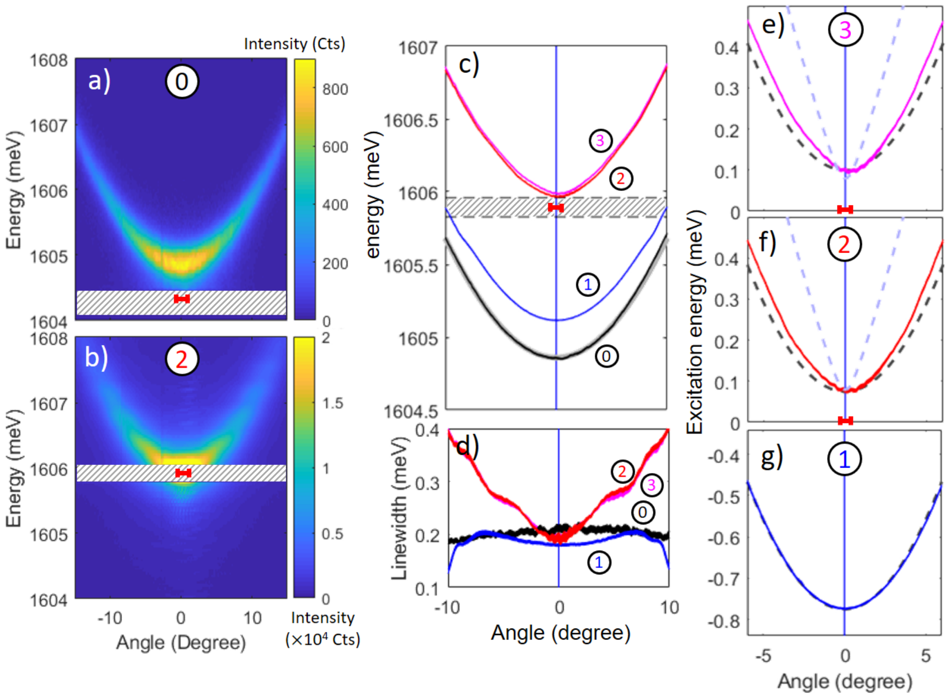

Supplementary Figure 4. **Excitation dispersion relation of Working point C** - Angle and energy resolved cross-polarized EPL measurements $I_e(\theta_p, \hbar\omega)$ in the free particle regime (a), and in the upper branch of $I_0(P)$ (b). The intensity is color-coded on a linear scale. The laser energy and angular spread is shown as a red segment. (c) Measured dispersion relations $\hbar\omega(\theta)$ as obtained from the numerical analysis (solid colored lines). The line thickness show the 95%-confidence interval of the experimentally determined dispersion relations. Four states of the fluids are shown labelled from 0 to 3: the free particle dispersion ('0'), a lower branch state of $I_0(P)$ ('1'), and two upper branch states ('2' and '3'). Panel (c) show the dispersions relations measured on absolute energy scale, while (e-g) show the dispersion relations '3' to '1' respectively measured from the condensate energy. The hatched rectangle in (a-c) show the spectral range rejected by the notch filter. (d) Measured excitation linewidth versus angle of states '0' to '3'. Panels (e-g) include two calculated limiting cases: the vanishing condensate fraction regime (black dashed line), and the regime of vanishing reservoir fraction (blue dashed line).

far above (100 meV) the free excitons states. In the latter case, the relaxation mechanism involves the same intermediate reservoir as in scenario (1) and (2) such that the thus obtained excitation spectrum $I_{\rm NR}(\hbar\omega)$ should be similar in shape to $I_e(\hbar\omega)$. The comparison is shown in Supplementary Figure 3.b at the same working point and temperature $T = 20\,$K: It turns out that these spectra are quite different, the one obtained under non-resonant excitation being significantly 'hotter'.

Scenario (3) is the most likely one in our experiment. We can support it further by checking that the condensate fluctuations are indeed excited by the thermal bath of acoustic phonons by measuring the polariton state occupancy $n(\hbar\omega)$ in the free particle regime (vanishing two body interactions), and compare it with a thermal distribution. $n(\hbar\omega)$ can be determined experimentally using the density of state $\rho(\hbar\omega)$ effectively contributing in the measurement, and $I_e(\hbar\omega)$ which is proportional to the fluctuation population times their linewidth. The measurement has been performed at $T = 30\,$K and the result is shown in log scale in Supplementary Figure 3.c. The excitations occupancy $n(\hbar\omega)$ obtained in this way agrees very well with a Maxwell-Boltzmann distribution at $T = 30\,$K. The distributions for $T = 27\,$K and $T = 33\,$K are shown as dashed lines. This result is a strong argument in favour of scenario (3).

SUPPLEMENTARY NOTE 4. WORKING POINT C

In order to support the generality of our observations, we have performed a full characterization of the polariton superfluid excitations at several other working points. Supplementary Figure 4 shows the characterization at working point C, in which the condensate is driven at normal incidence $\theta = 0°$. Supplementary Figure 4.a shows the raw EPL measurements in the red-shifted weak excitation regime, that we use to determine the reference free-particle dispersion, and Supplementary Figure 4.b shows the raw EPL measurement in a switched-up state. The hysteresis curve $I_0(P)$ is shown in Supplementary Figure 5.a. The analyzed dispersion relations are shown all together in and Supplementary Figure 4.c and individually in Supplementary Figure 4.e-g. In the latter case, the limiting cases theory (i) and (ii) discussed in

the main text are plotted for comparison as black and blue dashed line respectively. The extracted linewidth analysis is plotted as well in Supplementary Figure 4.d. As explained in the next section, the flat behaviour of $\hbar\gamma$ vs angle for the free-particle dispersion and the switched down states results from the absence of switching front, and of a spatial filter in the detection. The experimental parameters of WPC are $\delta = +0.7meV$, $T = 30$K, free particle linewidth at $k = 0$ $\hbar\gamma_c = 0.4$meV, and $\Delta = +1.04$meV.

SUPPLEMENTARY NOTE 5. CHARACTERISTICS OF THE CONDENSATE IN REAL SPACE

Spatial structure of the condensate

Owing to the Gaussian shape of the excitation spot the upper branch of the hysteresis is located within a round area at the center of the excitation spot, separated by a sharp switching front of diameter $D^{(2)}$ from the outer ring of polaritons, that are in a switched-down state. This is shown in Supplementary Figure 5 where the measured cross-polarized condensate at WPC is shown in real space for three different regimes: in the state labeled '1' in Supplementary Figure 5.b the whole fluid is in the lower branch of the hysteresis (which is shown in Supplementary Figure 5.a), and thus exhibits a gaussian intensity distribution. In Supplementary Figure 5.c the central part of the fluid labeled 'UB' has switched to the upper branch, while the outer rim labeled 'LB' remains in the lower branch. The switching front separating the two is shown as a dashed orange line. At higher intensity (state '3' in Supplementary Figure 5.d), the diameter of the switching front slightly increases, i.e. moves further towards the edges of the fluid.

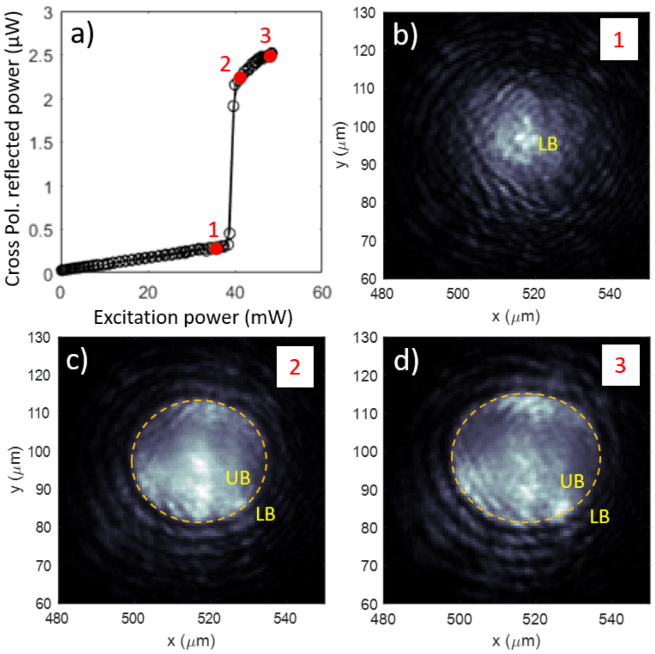

Supplementary Figure 5. **condensate spatial structure** - (a) Measured hysteresis curve $I_0(P)$ of the cross-polarized reflectivity for a working point with parameters identical to WPA. The cross-polarized condensate density is shown in real space for the states '1' (b), '2' (c) and '3' (d) marked on the hysteresis curve. 'LB' ('UB') stands for lower (upper) branch of $I_0(P)$. The dashed orange line shows the circular switching front separating the 'LB' and the 'UB' areas.

Influence on the measured linewidth $\hbar\gamma(\theta)$ in the high-density regime

The analysis of the raw measurement $I_e(\theta,\omega)$ also gives us access to the excitations spectral linewidth $\hbar\gamma$. The results are shown in Supplementary Figure 6 for both WPA (a) and WPB (b) together with the results of the full theory calculation. In the low excitation regime, (solid black line labelled '0' in WPA), the measured linewidth is essentially angle-independent. This is expected as over such a small angular range, the excitonic and photonic fraction hardly change vs θ: i.e. the kinetic energy increase as compared to the Rabi splitting is small (1 meV increase between 0° and 10°). For the measurements of $\hbar\gamma(\theta)$ in states '1' (switched-down state), '2', and '3' (switched-up states), we have inserted a circular aperture of diameter $D^{(1)}$ in the detection path that filters out the outer area of the excitation spot.

For the measurement of $\hbar\gamma(\theta)$ in states '2' and '3', both the finite size of the 'UB' area in real space, and the use of a circular aperture filter have a significant influence: in both cases, the excitations of the condensate have a finite transit time either throughout the circular aperture of the detection or within the switched up area, depending on which is the smallest. This transit time is fixed by the excitation group velocity $\tau(k) = D^{(1,2)}/v_g(\theta)$, which is a decreasing function of θ, regardless of the detailed nature of the excitation. For the switched down state shown in Supplementary Figure 5.a, there is no switching front such that the circular aperture of diameter $D^{(1)} = 40\,\mu$m actually fixes the increasing rate of $\bar{\gamma}$ versus θ. This is the case of state '1' in Fig.36.a in the main text. For states labeled '2' and '3' in Supplementary Figure 6.a and Supplementary Figure 6.b, the switching front diameters $D^{(2)}$ are comparable and close to $D^{(1)} = 40\,\mu$m. As a result, the increase of $\hbar\gamma$ versus θ reflects the presence of the switching front, and can thus be considered as a characteristic feature of a locally switched-up polariton fluid driven by a Gaussian spot.

While our full theory is translational invariant, we can still mimic this effect with it by adding a numerical spatial filter of diameter $D^{(2)}$ to the results. The result is shown is Supplementary Figure 6 as the light solid lines. We see that while the increasing linewidth versus θ is indeed reproduced, it is underestimated. Other sources of nonlinear broadening are likely to contribute for increasing excitations energy, as the reservoir becomes highly

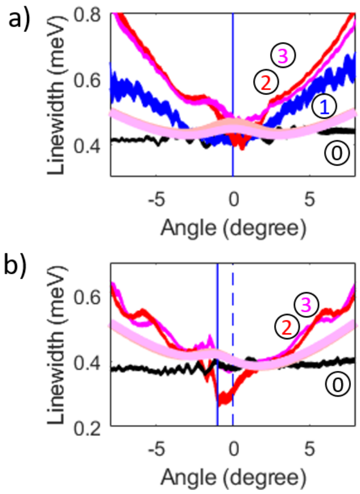

Supplementary Figure 6. **Linewidth versus momentum** - Measured excitations linewidth $\hbar\gamma(\theta)$ vs Angle, as obtained from the numerical analysis of the EPL (narrow solid lines) for WPA (a) and WPB (b). The full vectorial theory is shown as the thick solid lines. For the measurement data, the line thickness show the 95%-confidence interval. The same labelling convention is used as in Fig.3 of the main text. The solid vertical line show the laser excitation incidence angle, while the dashed one shows the zero incidence reference ($\theta_\mathrm{p} = 0°$)

populated (not accounted for in the present model).

Influence of the Gaussian spot on the dispersion relation of the excitations $\omega_\mathrm{e}(\theta)$

The density profiles shown in Supplementary Figure 5.c,d display two types of inhomogeneities: an overall modulation due to the pump Gaussian intensity profile, and random fluctuations due to in-plane disorder. We discuss here how the Gaussian spot may influence the dispersion relation, and in the next section the influence of disorder.

This problem is difficult to tackle in the framework of the full theory: the specific shape of the cloud strongly depends on the bistability features and, in turn, on the details of the switch-off jump at the edge of the cloud. However, we can take the simplifying assumptions of the local density approximation (LDA), which is reasonable, if not exact, considering the large size of the Gaussian spot ($\sigma_r = 25\,\mu$m radius).

We thus assume a pure, scalar polariton condensate for which the excitations dispersion relation $\omega_\mathrm{bog}(k_\parallel, \omega, n)$ is given by Eq.(2) of the main text. The pump spot has a bidimensionnal Gaussian intensity profile: $I_\mathrm{P}(r) = I_0/(\pi\sigma_r^2)\exp\left[-(r^2/\sigma_r^2)\right]$. The local polariton density $n(r)$ is derived within the LDA from the steady-state GGPE equation $I_\mathrm{P}(n) = n\left[(\Delta + gn)^2 + (\gamma/2)^2\right]$.

Using the experimental parameters $\hbar\Delta = -0.79\,$meV, and $\hbar\gamma = 0.4\,$meV (WPA), we obtain the hysteretic plot $I_\mathrm{P}(n)$ shown in Supplementary Figure 7.a, and the density distribution $n(r)$ (Supplementary Figure 7.b). This density profile is in qualitative agreement with the measured one shown in Supplementary Figure 5.c,d in terms of its 'flatness' in the switched-up area (excluding the fluctuations due to the weak disorder). Assuming a Lorentzian lineshape, the inhomogeneous spectral function $F(k,)$ is obtained in the LDA approximation as

$$F(k_\parallel, \omega) = \iint_f \mathrm{d}x\mathrm{d}y\, n(r)\mathcal{L}\left(\omega, \omega_\mathrm{bog}\left[k_\parallel, \omega, n(r)\right], \gamma\right), \tag{7}$$

where $n(r)\mathrm{d}x\mathrm{d}y$ is proportional to the local intensity $\mathrm{d}I(x,y)$, $\mathcal{L}(\omega, \omega_0, \gamma)$ is a Lorentzian lineshape centered at ω_0 and of linewidth (FWHM) γ, and f is the spatial filter (circular aperture) which is centered on the switched-up area, with a smaller diameter in order to reject the switching front separating the two regions, like in the ex-

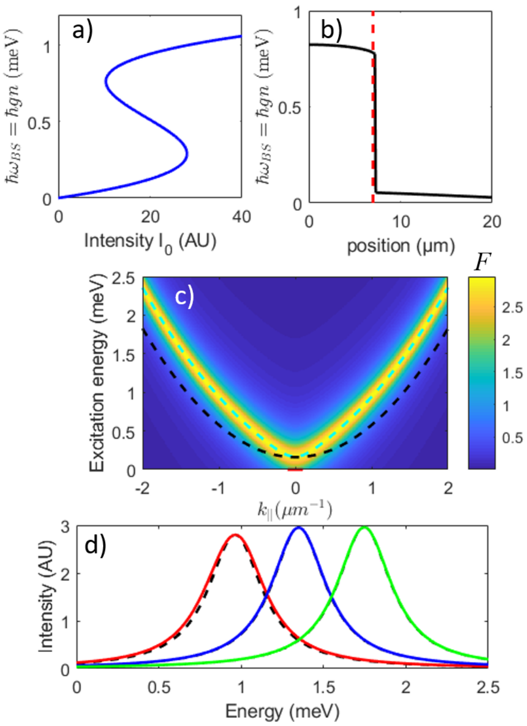

Supplementary Figure 7. **influence of the Gaussian spot on the dispersion relation** - a) calculated $n(I_0)$ for $\hbar\Delta = -0.79\,$meV, and $\hbar\gamma = 0.4\,$meV from the homogeneous steady-state GGPE. b) calculated Profile of the polariton density $n(r)$ in the LDA, for a Gaussian spot of size $\sigma_r = 25\,\mu$m and a peak intensity I_0 close enough to the switch down point in order to match the experimental blueshift (of WPA.2) $\hbar\omega_\mathrm{BS} = gn = 0.85$ meV. The dashed red line shows the spatial filter edge used in the calculation. c) Calculated inhomogeneous spectral function $F(k_\parallel, \omega)$ in the LDA. The cyan dashed line is the theoretical excitations dispersion relation of the homogeneous pure condensate. The black dashed line is the homogeneous rigid blueshift $\omega_\mathrm{RB}(k_\parallel)$. d) Cross-sections of $F(k_\parallel = k_j, \omega)$ for $k_j = 0, 0.6, 1\,\mu\mathrm{m}^{-1}$ in red blue and green respectively. The dashed line show the homogeneous lineshape $\mathcal{L}(\omega, \omega_0, \gamma)$ for comparison.

periment (red dashed line in Supplementary Figure 7.b). The calculated spectral function is shown in Supplementary Figure 7.c in colorscale. Like in the manuscript, we can compare it with the two limiting cases: *homogeneous* pure polaritons condensate dispersion relation (cyan dashed line), and rigidly blueshifted dispersion relation (black dashed line), both with a blueshift equal to the experimental one ($\hbar\omega_{\mathrm{BS}} = 0.85\,\mathrm{meV}$, WPA.2). We see very clearly that in spite of the inhomogeneity from the Gaussian spot, the fit with the pure condensate limit is nearly perfect. This is in contradiction with the measurement, in which the speed of sound is found twice too low as compared to the homogeneous pure condensate limit. This is far too large a deviation, even in the local density approximation.

Note that the LDA being an approximation, we tested parameters slightly different from the experimental ones in terms of linewidth and laser detuning Δ, for which the switched up density distribution is less flat. The deviation between the homogeneous dispersion relation and the inhomogeneous spectral function remained also largely negligible.

This analysis provides another relevant insight. In spite of using a Gaussian spot for the excitations, we see that the hysteretic behavior offers a 'flattening' of the polaritons density, which limits the inhomogeneous broadening of the excitations. This is also true in the case where a reservoir is involved. In essence, the nonlinearity acts as a spatial 'tophat' filter for the Gaussian pump spot, a strategy often used in optical spectroscopy to mimic a quasi-homogeneous spot intensity distribution. The positive influence of this flattening effect is visible (within the LDA) in Supplementary Figure 7.d, where inhomogeneous spectra $F(k_{\|} = k_j, \omega)$ for three values of k_j are plotted (color lines) and compared with the homogeneous lineshape $\mathcal{L}(\omega, \omega_0, \gamma)$ (black dashed line). In spite of the Gaussian excitation, we see a vanishing deviation between the two. This is in agreement with the experimentally measured linewidth shown in Supplementary Figure 6. Indeed, if we look at the excitation spectrum linewidth at $\theta = 0$, for which the effect of the spatial filter is minimum (due to vanishing group velocity), we see that in spite of a total blueshift of $0.85\,\mathrm{meV}$ (Supplementary Figure 6.a red line, $\theta = 0°$), the measured linewidth of $0.4\,\mathrm{meV}$ remains unchanged as compared to that in the non-interacting regime (Supplementary Figure 6.a black line, $\theta = 0°$), within the experimental uncertainty. This is a solid indication that, thanks to the nonlinear 'flattening' of the density, the Gaussian spot shape has a negligible influence on the excitations lineshape and on the dispersion relation shape.

Influence of disorder on the dispersion relation of the excitations $\omega_{\mathrm{e}}(\theta)$

The darker and brighter patches observed in Supplementary Figure 5.c,d are attributable to a local disorder

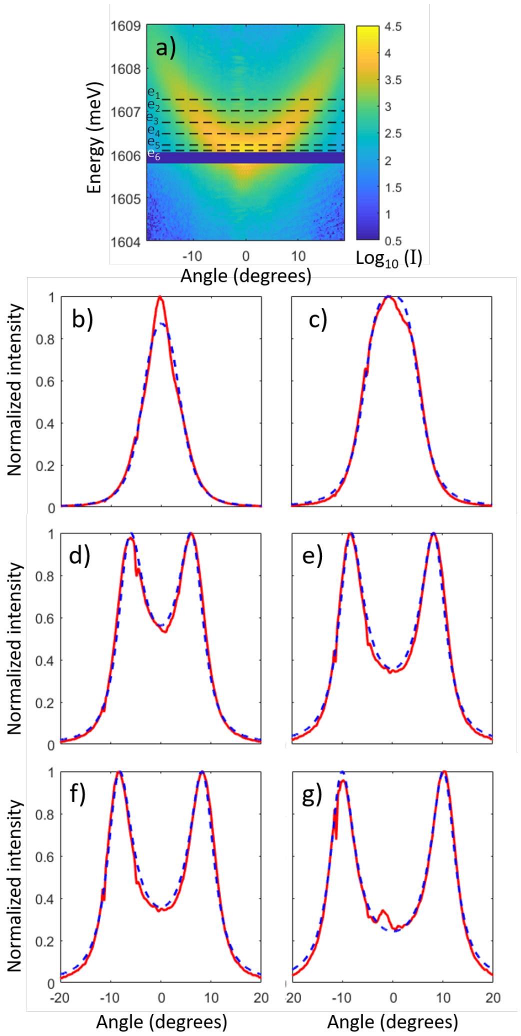

Supplementary Figure 8. **Angular cross-sections** - a) Raw measurement of $\log_{10}[I_{\mathrm{e}}(\theta, \hbar\omega)]$ of WPA point '3' (color scale). Experimental (red line) and theoretical (dashed blue line) horizontal cross sections $I_{\mathrm{e}}(\theta, e_i)$ are shown for $e_6 = 1606.09\,\mathrm{meV}$ (b), $e_5 = 1606.22\,\mathrm{meV}$ (c), $e_4 = 1606.48\,\mathrm{meV}$ (d), $e_3 = 1606.74\,\mathrm{meV}$ (e), $e_2 = 1607.01\,\mathrm{meV}$ (f), qnd $e_1 = 1607.27\,\mathrm{meV}$ (g). The small peak visible in panels b,c and g around $\theta = -2°$ is a residue from the pump laser.

of weak amplitude (as compared to the linewidth) and correlation length in the $3-5\,\mu\mathrm{m}$ (cf. Fig.2.b in the main text). Again, they are of unknown relative amplitude with respect to the total condensate density. Owing to the relatively small correlation length, the LDA approximation is probably not good enough to analyze their influence on the excitation dispersion relation. However, a disruptive disorder should result in (i) a significant broadening and a possible asymmetry of the measured spectra $I(\theta = \theta_j, \omega)$, and (ii) the signatures of localization: spectrally narrow states, broad in momentum space, and flat in dispersion.

We have looked for both signatures in the experimental data and found none of them. Measured spectra

$I(\theta=\theta_j,\omega)$ are shown in detail in Supplementary Figure 2.b, and exhibit no sharp peaks indicative of localization within the experimental uncertainty. We have also looked at cross-sections at fixed energies $I(\theta,\omega=\omega_j)$ (red lines in Supplementary Figure 8), and compare them with the spectral function of the free-polariton dispersion $f_{\rm p}(\theta,\omega=\omega_j)$ (dashed blue lines in Supplementary Figure 8). While this is obviously an approximate model, it is sufficient to check whether some anomalous broadening in momentum space are present in the experimental peaks. The linewidths used in this simple comparison are extrapolated from the table in section II of the SI. Very small corrections to the cross-section energy are applied in order to match angular-peaks from the free-polariton model and the experiment. It is clear from this comparison that we do not see any momentum broadening or signatures of localization.

SUPPLEMENTARY NOTE 6. STATISTICAL ANALYSIS OF THE CONDENSATE FRACTION AND RENORMALIZED SPEED OF SOUND

The comparisons between the theoretical dispersion relations and the measured ones, are shown quantitatively in Supplementary Figure 9 using the normalized deviation $R^2(\rho)=\sum_i[\omega_{\rm exp}(k_i)-\omega_{\rm th}(\rho,k_i)]^2/\sum_i\omega_{\rm exp}(k_i)^2$. Supplementary Figure 9 plots $r^2(\rho)=\min(R^2)/R^2(\rho)$ where

$$\rho=\frac{n_c}{n_{\rm R}+n_c}=\frac{\gamma_{\rm R}}{\gamma_{\rm in}+\gamma_{\rm R}}, \tag{8}$$

is the condensate fraction, $n_c=n_x+n_y=|\psi_x^s|^2+|\psi_y^s|^2$, $\gamma_{\rm R}=1.6\,\mu$eV as extracted from the decay time found in Supplementary Figure 1, and $\gamma_{\rm in}$ is the actual fit free parameter. The closer to r^2 is to 1, the better the agreement. The full width at half maximum of $r^2(\rho)$ provides an estimate of its 1σ-confidence interval. The line connecting the dots in Supplementary Figure 9 is a guide for the eye. The thus determined confidence intervals has been used in Fig.3 of the main text, where the theoretical dispersion plots is obtained by plotting the two dispersion relations calculated at the two confidence interval boundaries, and by coloring the area that they delimit.

This analysis also gives an estimate of the two speeds of sound that characterizes the two cross-polarized Bogoliubov branches at the sonic point of the hysteresis. In the simplifying assumption that $n_y=0$, i.e $\rho=n_x/(n_x+n_{\rm R})$, they have the analytical expression given in eq.(33) and eq.(35). The results are shown in Supplementary Figure 9 (right axis), where the speeds of sound c_x and c_y normalized to the critical velocity $v_{\rm c}=\sqrt{\hbar\omega_{\rm BS}/m}$ are plotted alongside $r^2(\rho)$.

The results of this whole analysis are summarized in the following table.

WP	ρ	CI(ρ)	$c_x/v_{\rm c}$	$c_y/v_{\rm c}$
A.2	47%	[40%;53%]	43%	48%
A.3	45%	[38%;50%]	42%	46%
B.2	78%	[70%;85%]	61%	68%
B.3	68%	[60%;73%]	52%	57%

where 'CI' stands for 'confidence interval'.

SUPPLEMENTARY NOTE 7. ESTIMATE OF THE POLARITON-POLARITON INTERACTION STRENGTH

A key advantage of this work is that we can extract the relative contribution of polariton-polariton interaction $\hbar\bar{g}n$ to the total blueshifts $\hbar\omega_{\rm BS}=\hbar\bar{g}n+\hbar g_{\rm R}n_{\rm R}$. Thus for instance in WPA point 2, out of $\hbar\omega_{\rm BS}=0.85\,$meV, $\hbar\bar{g}n=0.15\,$meV come from polariton-polariton interaction.

However, in order to derive an actual value of the polariton-polariton interaction constant $g_{\rm T}$ (where $2\bar{g}=g_{\rm T}+g_{\rm S}$), we need additional information such as an absolute estimate of the polariton density n, or of the pump term $|F|^2$. Since we have a solid measurement of the latter in our $I(P)$ measurement, we can use it to infer $g_{\rm T}$.

Assuming the scalar limit of our model, in the homogeneous and steady state regime, we get that

$$\left[(-\hbar\Delta+\hbar g_{\rm eff}n)^2+\frac{\hbar^2\bar{\gamma}^2}{4}\right]n=|F|^2, \tag{9}$$

where $g_{\rm eff}=\bar{g}+g_{\rm R}\gamma_{\rm in}/\gamma_{\rm R}$ and $\bar{\gamma}=(\gamma_{\rm c}+\gamma_{\rm in})$. The input-output theory of polaritons [5] provide a relation between the intracavity polaritonic field generation rate $|F|^2$ and the external pump of power $P_{\rm in}$:

$$|F|^2=\frac{\hbar\gamma}{2}|C|^2f_{\rm p}(r_a)\frac{P_{\rm in}}{\hbar\omega_{\rm l}}\frac{10^6\hbar^2}{e^2}, \tag{10}$$

where $|F|^2$ is expressed in [meV.μm^2] units, $|C|^2$ is the photonic fraction, $P_{\rm in}$ is the laser power in Watts, $f_{\rm p}(r)$ is a normalized Gaussian distribution describing the spot in μm^{-2} units, and r_a is an average radial position within the spot such that the spatially averaged blueshift $\langle\hbar g_{\rm eff}n\rangle$ is that found in the experiment. In introducing $f_{\rm p}(r)$ and neglecting the kinetic term in the equation of motion above, we have made the implicit assumption of the slowly varying envelope. In order to determine r_a we would need to know the distribution $n(\mathbf{r})$. We do not know it, but we have a measurement of the radius r_t of the UB in real space shown in Supplementary Figure 5.c: $r_t=17.5\,\mu$m.

We thus consider two extreme situations: $r_a=0$ and $r_a=r_t$ as forming a first contribution to the error bar of $g_{\rm T}$. We then fit the theoretical hysteresis $\hbar\omega_{\rm BS}(P_{\rm in})$ derived from the above equations such that (i) the jump-up occurs at the experimental one: $P_{ju}=30.1\,$mW as shown in $I(P)$ in Fig.2.a, and (ii) the total blueshift

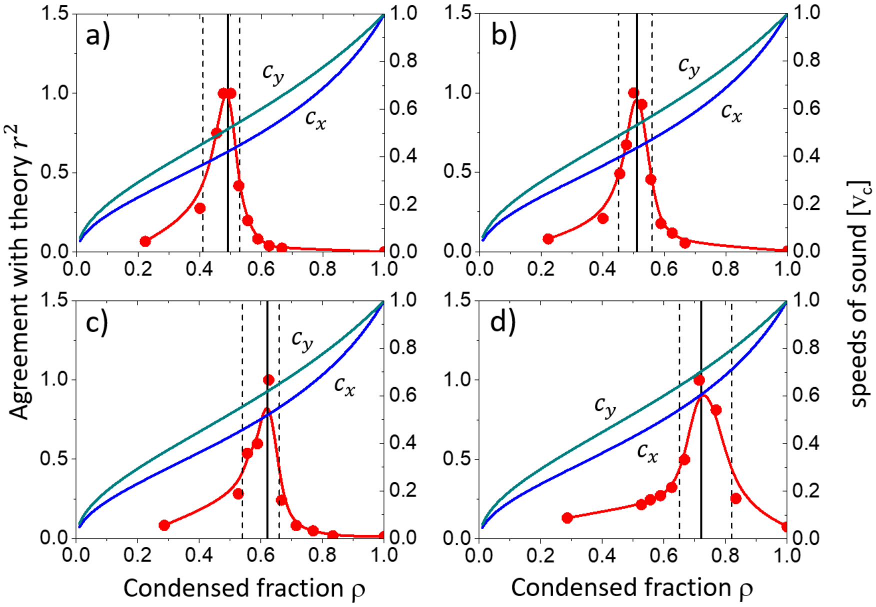

Supplementary Figure 9. **Statistical analysis of the condensate fraction by quantitative comparison between experiment and theory** - Agreement between the measured dispersion relation and the full vectorial theory, quantified as $r^2(\rho)$ =min$(R^2)/R^2(\rho)$ (red symbols), versus the condensate fraction $\rho = n_c/(n_R + n_c)$ for WPA.2 (a),WPA.3 (b), WPB.2 (c) and WPB.3 (d). The red solid line is a guide for the eye. The right axis show the calculated speed of sounds of the two cross polarized excitations c_x/v_c (blue line) and c_y/v_c (blue green line) versus ρ, in the limit where $n_y = 0$ (see text).

$\hbar g_{\rm eff} n$ matches the experimental one $\hbar\omega_{\rm BS} = 0.85\,$meV of point 2 at $r_a = 0$ or $r_a = r_t$.

In doing so, we find $\hbar g_{\rm T} = 8 \pm 2\mu$eV$\mu$m^2 in which the error bar is actually rather set by the larger uncertainty on $\gamma_{\rm in}/\gamma_{\rm R}$ than on r_a. This value is well in-line with the literature, with the advantage of ruling out for sure the reservoir contribution. We insist however that this method is approximate, as it doesn't take into account the spatial shape of the spot rigorously, which would require a much more involved modelization.

SUPPLEMENTARY NOTE 8. FULL VECTORIAL THEORY

Theoretical model

In order to model the experiment, we use a generalized Gross-Pitaevskii theory for the two polariton condensate wavefunctions $\psi_\sigma(\mathbf{r})$, corresponding to the two linear polarizations with $\sigma = x, y$ (pump-probe basis), coupled to a dark-exciton reservoir with density $n_{\rm R}$:

$$i\partial_t\psi_x = \left[\omega_{\rm LP}(\hat{\mathbf{k}}) - \frac{\alpha}{2}\cos(2\Theta) + \frac{g_{\rm T}+g_{\rm S}}{2}|\psi_x|^2 + g_{\rm T}|\psi_y|^2 + g_{\rm R}n_{\rm R} - i\frac{\gamma_{\rm c}+\gamma_{\rm in}}{2}\right]\psi_x - \frac{\alpha}{2}\sin(2\Theta)\,\psi_y - \frac{g_{\rm T}-g_{\rm S}}{2}\psi_x^*\psi_y^2 + F \tag{11}$$

$$i\partial_t\psi_y = \left[\omega_{\rm LP}(\hat{\mathbf{k}}) + \frac{\alpha}{2}\cos(2\Theta) + \frac{g_{\rm T}+g_{\rm S}}{2}|\psi_y|^2 + g_{\rm T}|\psi_x|^2 + g_{\rm R}n_{\rm R} - i\frac{\gamma_{\rm c}+\gamma_{\rm in}}{2}\right]\psi_y - \frac{\alpha}{2}\sin(2\Theta)\,\psi_x - \frac{g_{\rm T}-g_{\rm S}}{2}\psi_y^*\psi_x^2 \tag{12}$$

$$\partial_t n_{\rm R} = -\gamma_{\rm R}n_{\rm R} + \gamma_{\rm in}(|\psi_x|^2 + |\psi_y|^2) \tag{13}$$

where $\omega_{\rm LP}(\hat{\mathbf{k}}) = \omega_{\rm LP,0} - \frac{\hbar}{2m}\nabla^2$ and $\hat{\mathbf{k}} = -i\nabla$, with m the effective polariton mass, $\alpha \sim 0.1 \pm 0.05$ meV is a

birifringence splitting and $\Theta \simeq 19°$ is the angle between the natural cavity axis and the pump-probe x, y polarization basis. g_T and g_S are the triplet and singlet coupling constants respectively, and we take $g_S = -0.1\ g_T$ and $g_T > 0$. The pump field is $F(\mathbf{r},t) = F_0 e^{i\mathbf{k}_p\cdot\mathbf{r} - i\omega_p t}$. The other parameters are the reservoir filling rate γ_{in} from the condensate, the reservoir decay rate γ_R and the condensate radiative loss rate γ_c.

Steady-state

If the detuning $\Delta = \omega_p - \omega_{LP,0}$ is sufficiently large, the steady state solution $\{\psi_x^s, \psi_y^s, n_R^s\}$ of Eqs.(11-13) displays a bistable behaviour for ψ_x^s as a function of the pump intensity $|F_0|^2$, similar to the well-known solution in the single-polarization case [5], while for ψ_y^s the upper branch of the hysteresis is decreasing, due to the competing interactions with ψ_x^s.

Excitation spectrum

By linearizing Eqs.(11-13) for small deviations from the steady state, ie $\delta\psi_\sigma = \psi_\sigma - \psi_\sigma^s$, $\delta n_R = n_R - n_R^s$, we obtain the generalized Bogoliubov-De Gennes equations

$$i\partial_t \delta\vec{\psi}(\mathbf{r},t) = \hat{\mathscr{L}}(\hat{\mathbf{k}})\delta\vec{\psi}(\mathbf{r},t) \tag{14}$$

where $\delta\vec{\psi} = (\delta\psi_x, \delta\psi_x^*, \delta\psi_y, \delta\psi_y^*, \delta n_R)$ is the fluctuation vector. The Bogoliubov spectrum then consists in five eigenbranches $\omega(k)$ related by particle-hole symmetry. Supplementary Figure 10 show examples of the five eigenbranches visible in the direction cross-polarized with the pump, calculated with the parameters of WPA, and different pump strength $|F|^2$. The flat branch has a mostly reservoir character, while the four others have a mostly polaritonic character. Owing to the brifreingence characteristic ($\alpha = 0.1$ meV and $\Theta = 19°$) The red modes originates mostly from the x component (co-polarized with the laser drive), while the blue modes originate mostly from the y component (cross-polarized with the laser drive).

In order to account for the experimental observations, we perform a linear-response analysis for the field amplitude $\delta\vec{\psi} = (\delta\psi_x(\mathbf{k}), \delta\psi_x^*(-\mathbf{k}), \delta\psi_y(\mathbf{k}), \delta\psi_y^*(-\mathbf{k}), \delta n_R(\mathbf{k}))$

$$\delta\vec{\psi} = [\omega - \hat{\mathscr{L}}(\mathbf{k})]^{-1}\delta\vec{F} \tag{15}$$

as the response to a stochastic drive $\delta\vec{F} = (\delta F_x(\mathbf{k},\omega), -\delta F_x^*(-\mathbf{k},-\omega), \delta F_y(\mathbf{k},\omega), -\delta F_y^*(-\mathbf{k},-\omega), 0)$. Assuming that the main excitation mechanism is the coupling to acoustic phonons [6], one has $\delta F_\sigma(\mathbf{k},\omega) = \psi_\sigma^s \mathcal{T}(\mathbf{k},\omega)$, with $\mathcal{T}(\mathbf{k},\omega)$ a stochastic phonon field. After averaging over the random realizations of the phonon field and defining

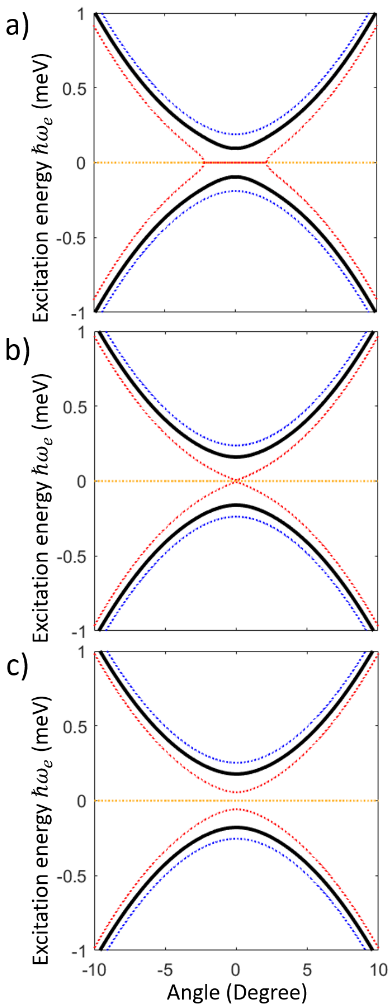

Supplementary Figure 10. **Dispersion relations of the excitations in the vectorial model** - The five theoretical branches obtained from Eq.(14) are shown as dotted lines. The mostly reservoir excitation branch is shown in orange. The mostly polaritonic excitation branches are shown in red (dominant x character) and blue (dominant y character). The normal (ghost) branches have a positive (negative) energy. The black line shows the theoretical dispersion as extracted from the calculated $I(\mathbf{k},\omega)$. The parameters are those of WPA: $\Delta = 0.79$ meV, $\Theta = \pi/9$, $\alpha = 0.1$ meV, $g_S = -0.1 g_T$, $\hbar\gamma_R = \hbar\gamma_i = 0.0016$ meV. Different pump strength are shown: (a) $|F|^2 \simeq |F_{ju}|^2$ (a), (b) $|F|^2 = 1.03|F_{ju}|^2$, and (c) $|F|^2 = 1.05|F_{ju}|^2$, where $|F_{ju}|^2$ is the jump-up pump strength.

$\chi_{ij}(\mathbf{k},\omega) = \left[\frac{1}{\omega - \hat{\mathscr{L}}(\mathbf{k})}\right]_{ij}$, the field intensity is

$$\langle\langle|\delta\psi_y(\mathbf{k},\omega)|^2\rangle\rangle_{ph} = |\chi_{31}\psi_x^s - \chi_{32}\psi_x^{s*} + \chi_{33}\psi_y^s - \chi_{34}\psi_y^{s*}|^2\ S_{ph}(\mathbf{k},\omega) \tag{16}$$

where S_{ph} is the phonon density of state, taken in this work as constant. Finally, in order to model the effect of the finite pump spot and of the iris, we multiply the polariton field by a spatial filter function f corresponding to a circular hole of diameter $35\mu m$, $\psi_y^{out}(\mathbf{r},t) =$

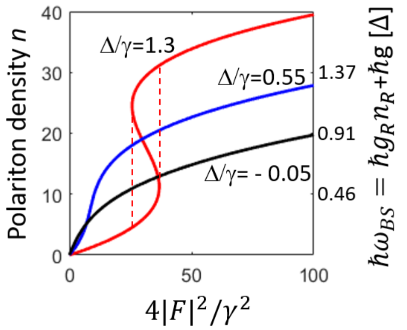

Supplementary Figure 11. **Hysteretic response of $n(|F|^2)$ in the scalar limit,** in three different conditions: $\Delta/\gamma = -0.05$ (black line), $\Delta/\gamma = 0.55$ (blue line), and $\Delta/\gamma = 1.3$ (red line). Only the latter exhibits a hysteretic behaviour. The horizontal axis is the unitless polariton generation rate normalized to the loss rate $4|F|^2/\gamma_c^2$.

$f(\mathbf{r})\psi_y(\mathbf{r},t)$; in the end, the measured Fourier space intensity is $I(\mathbf{k},\omega) = \int d\mathbf{k}'|f(\mathbf{k}-\mathbf{k}')|^2\langle\langle|\delta\psi_y(\mathbf{k}',\omega)|^2\rangle\rangle_{ph}$. $I(\mathbf{k},\omega)$ is our actual experimental observable. We thus apply to it the same numerical analysis as for the experimental one: we fit it with a single Lorentzian lineshape, in order to obtain a single theoretical dispersion relation (two if we accounts for positive and negative energy branches), to be compared with the experimental one. The result of this procedure is shown as a black line in Supplementary Figure 10.

Scalar limit of the model

In the main text, we take the scalar limit of the vectorial model as it is much simpler to discuss. It would be valid for instance under circular excitations, and co-polarized detection. Its expression reads

$$i\hbar\partial_t\psi = \Big[\hbar\omega_0 - \frac{\hbar^2}{2m}\nabla^2 + \hbar g|\psi|^2 + \hbar g_R n_R - i\frac{\hbar(\gamma+\gamma_{in})}{2}\Big]\psi + F(t) \quad (17)$$

$$\partial_t n_R = -\gamma_R\, n_R + \gamma_{in}|\psi|^2, \quad (18)$$

where m is the polariton effective mass, $F(t)$ is the resonant, spatially homogeneous laser drive, γ is the radiative loss rate, and the much slower capture rate of polaritons by the reservoir is γ_{in}. The polariton-polariton interaction energy is proportional to the scalar density $n = |\psi|^2$ with a coupling constant g, while the interactions energy between polaritons and the reservoir is fixed by $\hbar g_R n_R$.

The corresponding steady-state solution for the polariton density reads

$$\left[(-\hbar\Delta + \hbar g_{\rm eff} n)^2 + \frac{\hbar^2\bar{\gamma}^2}{4}\right] n = |F|^2, \quad (19)$$

where $g_{\rm eff} = g + g_R\gamma_{in}/\gamma_R$ and $\bar{\gamma} = (\gamma+\gamma_{in})$, and the total blueshift $\hbar\omega_{BS} = g_{\rm eff} n$. This function exhibits a hysteretic behaviour as soon as $\hbar\Delta \le \hbar\gamma\sqrt{3}/2$. Examples of steady-state polariton density n versus pump strength $|F|^2$ are shown in Supplementary Figure 11.

Derivation of the model

We provide here below the derivation of the Hamiltonian used to model the experiment.

Kinetic part

The cavity used in the experiment has some intrinsic birifringence. In the linear polarization basis $|s\rangle, |f\rangle$, associated to the axis of the cavity, the Hamiltonian for the lower polariton (LP) branch reads

$$H_{\rm bir} = \int d^2r\, \hat{\psi}_s^\dagger\left(\omega_{LP}(\hat{\mathbf{k}}) - \frac{\alpha}{2}\right)\hat{\psi}_s + \hat{\psi}_f^\dagger\left(\omega_{LP}(\hat{\mathbf{k}}) + \frac{\alpha}{2}\right)\hat{\psi}_f, \quad (20)$$

where the LP band is taken in the parabolic approximation $\omega_{LP}(\hat{\mathbf{k}}) = \omega_0^{LP} - \frac{\hbar}{2m}\nabla^2$, with m the effective polariton mass and the field operators $\hat{\psi}_s(\mathbf{r})$, $\hat{\psi}_f(\mathbf{r})$ destroy a boson with polarization s, f respectively at spatial position $\mathbf{r}$.

In our experiment the birefringence splitting is $\alpha \sim 0.1 \pm 0.05$ meV and the laser is pumped with a linear polarization x rotated by an angle $\Theta \simeq -19°$ with respect to the s axis, ie we have

$$\begin{pmatrix}\hat{\psi}_s\\ \hat{\psi}_f\end{pmatrix} = \begin{pmatrix}\cos\Theta & \sin\Theta\\ -\sin\Theta & \cos\Theta\end{pmatrix}\begin{pmatrix}\hat{\psi}_x\\ \hat{\psi}_y\end{pmatrix}, \quad (21)$$

so that the kinetic part of the Hamiltonian in the $|x\rangle, |y\rangle$ linear polarization basis reads

$$H_{\rm kin} = \int d^2r\, \begin{pmatrix}\hat{\psi}_x^\dagger & \hat{\psi}_y^\dagger\end{pmatrix}\left[\omega_{LP}(\hat{\mathbf{k}})\mathbb{I} + \frac{\alpha}{2}\begin{pmatrix}-\cos 2\Theta & -\sin 2\Theta\\ -\sin 2\Theta & \cos 2\Theta\end{pmatrix}\right]\begin{pmatrix}\hat{\psi}_x\\ \hat{\psi}_y\end{pmatrix}, \quad (22)$$

Two-body interactions

Using spin conservation, the polariton–polariton interaction is naturally written in the circular polarization basis $|\sigma_\pm\rangle = (|x\rangle \pm i|y\rangle)/\sqrt{2}$ as

$$H_{\rm int} = \frac{1}{2}\int d^2r\,\Big[\, g_T(\hat{\psi}_+^\dagger\hat{\psi}_+^\dagger\hat{\psi}_+\hat{\psi}_+ + \hat{\psi}_-^\dagger\hat{\psi}_-^\dagger\hat{\psi}_-\hat{\psi}_-) + 2g_S\hat{\psi}_+^\dagger\hat{\psi}_-^\dagger\hat{\psi}_+\hat{\psi}_-\Big] \quad (23)$$

where $\hat{\psi}_{\pm}(\mathbf{r})$ denote the field operators for $\sigma_{\pm}$ circular polarizations respectively. In the simulations we take the values $g_{\rm S}/g_{\rm T} \sim -0.1$, $g_{\rm T} > 0$, in agreement with known properties of III-V semiconductor microcavities [7, 8].

The full vectorial Hamiltonian for the polariton field then reads

$$H_0 = H_{\rm kin} + H_{\rm int} + \int d^2r \left[F(\mathbf{r},t)\hat{\psi}_x^{\dagger}(\mathbf{r}) + F^*(\mathbf{r},t)\hat{\psi}_x(\mathbf{r})\right] \tag{24}$$

where $F(\mathbf{r},t)$ is the laser pump. Notice that in the experiment only the x-polarization component of the polariton field is pumped.

Reservoir population, incoherent losses and cavity losses

We consider an excitonic reservoir coupled to the polariton density as in [9], density coupled to the condensate. We also model the polariton radiative losses from the cavity by introducing a loss rate constant $\gamma_{\rm c}$ for both polariton polarizations.

Using the above results in the mean-field approximation for the polariton fields, one readily derives the Gross–Pitaevskii equations given in Eq.(3) in the main text, as well as the equation for the reservoir (Eq.(4) of the main text).

Polariton-phonon interactions

Finally, we describe the coupling of polaritons to acoustic phonons by a deformation-potential interaction via the Hamiltonian [10]

$$H_{\rm pol-phon} = \sum_{\mathbf{q},q_z} G_{\mathbf{q},q_z}(b_{\mathbf{q},q_z} - b^{\dagger}_{-\mathbf{q},-q_z})\rho_{\mathbf{q}} \tag{25}$$

where $G_{\mathbf{q},q_z}$ is the acoustic phonon-polariton coupling strength taking into account the anisotropy due to the presence of the quantum well confinement in the z direction [6, 10], $b_{\mathbf{q},q_z}$ is the phononic field operator and $\rho_{\mathbf{q}}$ is the density fluctuation of the polariton condensate with cavity in-plane momentum $\mathbf{q}$.

The above Hamiltonian yields a stochastic phonon field in the Gross-Pitaevskii equation acting on both polarization components $\sigma = x, y$ of the polariton condensate according to $(\sum_{\mathbf{q}}[\mathcal{T}(\mathbf{q},t) + \mathcal{T}^*(-\mathbf{q},t)]e^{i\mathbf{q}\cdot\mathbf{r}})\psi_\sigma(\mathbf{r})$, where

$$\langle\langle \mathcal{T}^*(\mathbf{q},t)\mathcal{T}(\mathbf{q}',t')\rangle\rangle_{ph} = \sum_{q_z} |G_{\mathbf{q},q_z}|^2 \delta_{\mathbf{q},\mathbf{q}'} n(\omega_{\mathbf{q},q_z})\delta(t-t'), \tag{26}$$

with $\langle\langle\cdots\rangle\rangle_{ph}$ being the average over the noise realizations, $n(\omega_{\mathbf{q},q_z}) = 1/(e^{\hbar\omega_{\mathbf{q},q_z}/k_{\rm B}T} - 1)$, T the temperature and $\omega_{\mathbf{q},q_z}$ the acoustic phonon dispersion. Notice that the sum over q_z ensures a non-zero matrix element at small $\mathbf{q}$ even if $|G_{\mathbf{q},q_z}|^2$ vanishes for $|\mathbf{q}|, q_z \to 0$. The use of a constant power spectral density $S(\mathbf{q},\omega)$ is equivalent to the high temperature approximation of Eq. (26).

As described in the methods section in the main text, within the linear response theory we treat the field $\mathcal{T}(\mathbf{q},\omega)$ to order one in perturbation theory (so that ψ_σ enters at order 0 in δF, with consequent decoupling of the Fourier components), and calculate the linear response of the polariton condensate to it.

Bogoliubov excitations excluding birefringence

We derive in this section the analytical expression for the speed of sound given in the main text and provide an analysis of the nature of Bogolubov excitations. For this purpose we consider the limit where the birifringence parameter α is set to zero.

For $\alpha = 0$ the steady state features $\psi_y^s = 0$, while ψ_x^s is given by the standard bistability condition [5] with renormalized nonlinear coupling strength $g_{\rm eff} = \frac{g_{\rm T}+g_{\rm S}}{2} + \frac{g_{\rm R}\gamma_{\rm in}}{\gamma_{\rm R}}$ and the steady-state reservoir is given by $n_{\rm R}^s = \frac{\gamma_{\rm in}}{\gamma_{\rm R}}|\psi_x^s|^2$. As a consequence of the steady-state condition, the Bogoliubov equations for the excitations for the condensate with y polarization are decoupled from those of the condensate with x polarization and the reservoir:

$$\begin{aligned} i\partial_t\delta\psi_x = &\left[-\Delta - \frac{\hbar}{2m}\nabla^2 + 2\bar{g}|\psi_x^s|^2 + g_{\rm R}n_{\rm R}^s - i\frac{\bar{\gamma}}{2}\right]\delta\psi_x \\ &+ \bar{g}{\psi_x^s}^2\delta\psi_x^* + g_{\rm R}\psi_x^s\delta n_{\rm R} \end{aligned} \tag{27}$$

$$\partial_t\delta n_{\rm R} = -\gamma_{\rm R}\delta n_{\rm R} + \gamma_{\rm in}(\psi_x^s\delta\psi_x^* + {\psi_x^s}^*\delta\psi_x), \tag{28}$$

and

$$\begin{aligned} i\partial_t\delta\psi_y = &\left[-\Delta - \frac{\hbar}{2m}\nabla^2 + g_{\rm T}|\psi_x^s|^2 + g_{\rm R}n_{\rm R}^s - i\frac{\bar{\gamma}}{2}\right]\delta\psi_y \\ &- g_{\rm d}{\psi_x^s}^2\delta\psi_y^*, \end{aligned} \tag{29}$$

where $\Delta = \omega_{\rm p} - \omega_{LP}^0$, $\bar{g} = (g_{\rm T} + g_{\rm S})/2$, $g_{\rm d} = (g_{\rm T} - g_{\rm S})/2$ $\bar{\gamma} = (\gamma_{\rm c} + \gamma_{\rm in})$. The corresponding 5×5 Bogoliubov matrix $\mathscr{L}(\hat{\mathbf{k}})$ separates into two block matrices, namely a $3{\times}3$ part for the x-polarized condensate and reservoir, and a 2×2 part for the y-polarized condensate.

We start discussing the $3{\times}3$ part:

$$\mathscr{L}(\mathbf{k})|_{x,{\rm R}} = \begin{pmatrix} \eta_x(\mathbf{k}) - i\frac{\bar{\gamma}}{2} & \bar{g}{\psi_x^s}^2 & g_{\rm R}\psi_x^s \\ -\bar{g}{\psi_x^s}^{*2} & -\eta_x(-\mathbf{k}) - i\frac{\bar{\gamma}}{2} & -g_{\rm R}\psi_x^* \\ i\gamma_{\rm in}\psi_x^s & i\gamma_{\rm in}{\psi_x^s}^* & -i\gamma_{\rm R} \end{pmatrix}, \tag{30}$$

where $\eta_x(\mathbf{k}) = -\Delta + k^2/2m + 2\bar{g}|\psi_x^s|^2 + g_{\rm R}n_{\rm R}$. The structure of the above matrix is characterized by particle-hole symmetry, ie

$$\mathscr{P}\mathscr{L} = -\mathscr{L}\mathscr{P} \tag{31}$$

where

$$\mathscr{P} = \mathscr{K}\begin{pmatrix} 0 & 1 & 0 \\ 1 & 0 & 0 \\ 0 & 0 & 1 \end{pmatrix} \tag{32}$$

and $\mathscr{K}$ stands for complex conjugation. This symmetry implies $\mathscr{L}\mathscr{P}|\omega\rangle = -\omega^*\mathscr{P}|\omega\rangle$, so that $\mathscr{P}$ links pairs of eigenvectors. Since the size of the matrix is three and the system is parity invariant, this analysis allows us to immediately conclude that one eigenvlue is purely imaginary, and we attribute it to the reservoir branch. The remaining two eigenvalues, corresponding to the particle and hole branches, take the form $\omega^{\pm}_{\rm X}(\mathbf{k}) = \pm\epsilon(k) - i\frac{\gamma(k)}{2}$.

In the case $\Delta = \hbar\omega_{\rm BS}$, where $\hbar\omega_{\rm BS} = \bar{g}|\psi_x|^2 + g_{\rm R}n_{\rm R}$, the excitation spectrum has a linear, gapless dispersion. By solving the eigenvalue problem at long wavelength we obtain $\omega^{\pm}_{\rm X}(\mathbf{k}) = \pm c_x k - i\bar{\gamma}/2$ with

$$c_x^2 = \frac{\hbar\omega_{\rm BS}}{m} - \frac{\bar{\gamma}}{(\bar{\gamma}-2\gamma_{\rm R})}\frac{g_{\rm R}n_{\rm R}}{m}, \quad (33)$$

holding for $c_x^2 > 0$. In the limit $\gamma_{\rm R} \ll \bar{\gamma} = (\gamma_{\rm c} + \gamma_{\rm in})$, corresponding to the experimental conditions where the reservoir reacts slowly to fluctuations in the condensate, we obtain $c_x^2 = \hbar\omega_{\rm BS}/m - g_{\rm R}n_{\rm R}/m = \bar{g}|\psi_x^s|^2/m$, indicating that only the energy due to the condensate contributes to the speed of sound, while the energy stored in the dark excitonic reservoir acts just as a global energy shift. In the adiabatic limit $\gamma_{\rm R} \gg \bar{\gamma}$, when the reservoir responds instantaneously to the condensate one finds that both reservoir and condensate contribute to the speed of sound, ie $c_x^2 = \hbar\omega_{\rm BS}/m$.

In the y-polarization sector the Bogolubov matrix reads

$$\mathscr{L}(\mathbf{k})\big|_y = \begin{pmatrix} \eta_y(\mathbf{k}) - i\frac{\bar{\gamma}}{2} & g_{\rm d}\psi_x^{s\,2} \\ -g_{\rm d}\psi_x^{s\,*2} & -\eta_y(-\mathbf{k}) - i\frac{\bar{\gamma}}{2} \end{pmatrix}, \quad (34)$$

where $\eta_y(\mathbf{k}) = -\Delta + k^2/2m + g_{\rm T}|\psi_x^s|^2 + g_{\rm R}n_{\rm R}$. The condition for having gapless Bogoliubov excitations in the above equation is $-\Delta + g_{\rm T}|\psi_x^s|^2 + g_{\rm R}n_{\rm R}^s = g_{\rm d}|\psi_x^s|^2$. Interestingly, this coincides with the condition $\Delta = \hbar\omega_{\rm BS}$ required for having a gapless x branch. At low momenta the dispersion relation of the y branch reads $\omega_y^{\pm}(\mathbf{k}) = \pm c_y k - i\bar{\gamma}/2$, with

$$c_y^2 = \frac{g_{\rm d}(\mu_y - g_{\rm R}n_{\rm R})}{m} = \frac{g_{\rm T}-g_{\rm S}}{2}\frac{|\psi_x^s|^2}{m} \quad (35)$$

where $\mu_y = g_{\rm T}|\psi_x^s|^2 + g_{\rm R}n_{\rm R}$. Notice that our choice of parameters $g_{\rm T} > 0$, $g_{\rm S}/g_{\rm T} = -0.1$ implies that the y branch of the phonon dispersion lies at higher energy than the x branch in all regimes, which implies that $c_y > c_x$.

Next, we analyze how the reservoir influences the nature of the eigenmodes of the Bogolubov excitations. Using Eq.(28) above we get the relation between the variation of the condensate density and the one of the reservoir density,

$$\frac{\delta n_{\rm R}}{\delta(|\psi_x|^2 + |\psi_y|^2)} = \frac{\gamma_{\rm in}}{\gamma_{\rm R} - i\omega_a(\mathbf{k})}, \quad (36)$$

that holds for any eigenmode. Fig. 12 shows the argument of this quantity for the case of the $a = x$ branch, at $\alpha = 0$ and at the point with gapless excitation spectrum. We notice that at small wavevector $|{\rm Re}[\omega_x(k)]| \ll |{\rm Im}[\omega_x(k)]| \simeq \bar{\gamma}$ the density fluctuations of the condensate and of the reservoir are in phase opposition. This is in agreement with the fact that the excitation branch $\omega_x(\mathbf{k})$ is a Goldstone mode, so at small k the system tries to keep constant density by making $\delta n_{\rm R}$ and $\delta|\psi_x|^2$ oscillate with a relative phase π to compensate each other. At large momentum $|{\rm Re}[\omega_x(k)]| \gg |{\rm Im}[\omega_x(k)]|$ instead, $\delta n_{\rm R}$ follows $\delta|\psi_x|^2$ in quadrature of phase, with the condensate density fluctuations driving the reservoir density ones. Given the complex nature of $\omega_a(\mathbf{k})$, the transition between the two regimes occurs when the real part of the Bogoliubov energy ${\rm Re}[\omega_a(\mathbf{k})]$ is of the order of the loss rate $\bar{\gamma}$.

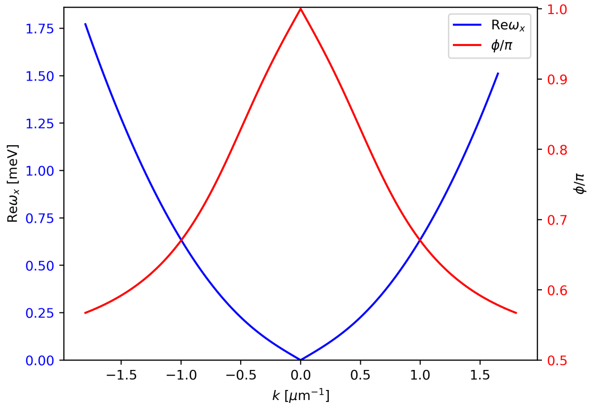

Supplementary Figure 12. **relative phase between the condensate and the reservoir excitations in the eigenbranch** $\omega_{\rm X}(k)$ - Blue, left axis: real part of $\omega_{\rm X}(\mathbf{k})$. The phase difference $\phi = \arg\delta n_{\rm R}/\delta|\psi_x|^2$ along the x positive eigenbranch is shown in red, (right axis). For the calculation we have chosen $\Delta = \hbar\omega_{\rm BS} = 0.8$ meV, $\alpha = 0$ (so that the y polaritons are decoupled), $\hbar^2/m = 1.0$ meVμm^2, $g_{\rm R}/\bar{g} = 4.0$, $\gamma_{\rm R} = 0.0016$ meV, $\bar{\gamma} = 0.4$ meV, $E_{\rm c}/E_{\rm tot} = 0.2$. This last condition fixes $\gamma_{\rm in} = 0.0016$ meV

Finally, we extend the above analysis to the case of finite in-plane momentum of excitation ie for $\mathbf{k}_{\rm p} \neq 0$. The steady-state solution takes the form $\psi_x^s(\mathbf{r}) = |\psi_x^s|\, e^{i\mathbf{k}_{\rm p}\cdot\mathbf{r}}$. In the case $\alpha = 0$ the Bogoliubov matrix Eq.(30) for the x-polarization condensate coupled to the reservoir is modified to [11]

$$\mathscr{L}_{\mathbf{k}_{\rm p}}(\mathbf{k})\big|_{x,R} = \begin{pmatrix} \eta_x(\delta\mathbf{k}+\mathbf{k}_{\rm p}) - i\frac{\bar{\gamma}}{2} & g\psi_x^{s\,2} & g_{\rm R}\psi_x^s \\ -g\psi_x^{s\,*2} & -\eta_x(\delta\mathbf{k}-\mathbf{k}_{\rm p}) - i\frac{\bar{\gamma}}{2} & -g_{\rm R}\psi_x^{s\,*} \\ i\gamma_{\rm in}\psi_x^s & i\gamma_{\rm in}\psi_x^{s\,*} & -i\gamma_{\rm R} \end{pmatrix}. \quad (37)$$

where $\delta\mathbf{k} = \mathbf{k} - \mathbf{k}_{\rm p}$ is the momentum measured in the fluid reference frame.

Interestingly, in this case the inversion symmetry is broken, and the reservoir excitation branch acquires a

real part (while particle–hole symmetry only requires $\mathscr{P}|\delta\mathbf{k},\omega\rangle = |-\delta\mathbf{k},-\omega^*\rangle$). In particular, at the equilibrium point $\Delta - \frac{\hbar^2 k_p^2}{2m} - \hbar\omega_{BS} = 0$, the real part of the reservoir eigenvalue is of order three in $|\delta\mathbf{k}|$ for small $|\delta\mathbf{k}|$, while $\omega_X(\mathbf{k},\mathbf{k}_p)$ in the same regime is modified to first order in $|\delta\mathbf{k}|$ only by the usual Doppler shift [11]: at given $\hbar\omega_{BS}$ we have $\omega_X(\mathbf{k},\mathbf{k}_p) = \frac{\mathbf{k}_p\cdot\delta\mathbf{k}}{m} + \omega_X(|\delta\mathbf{k}|,0)$.

[1] Daniele Bajoni, Pascale Senellart, Aristide Lemaître, and Jacqueline Bloch, "Photon lasing in GaAs microcavity: Similarities with a polariton" *Phys. Rev. B* **76**, 201305(R) (2007).

[2] S. Klembt, E. Durupt, S. Datta, T. Klein, A. Baas, Y. Léger, C. Kruse, D. Hommel, A. Minguzzi, and M. Richard, "Exciton-Polariton Gas as a Nonequilibrium Coolant" Phys. Rev. Lett. **114**, 186403 (2015)

[3] P. Borri, W. Langbein, U. Woggon, J. R. Jensen, and J. M. Hvam, "Microcavity polariton linewidths in the weak-disorder regime" Phys. Rev. B **63**, 035307 (2000)

[4] Vincenzo Savona, "Effect of interface disorder on quantum well excitons and microcavity polaritons" J. Phys.: Condens. Matter **19** 295208 (2007)

[5] I. Carusotto, and C. Ciuti, "Quantum fluids of light", Rev. Mod. Phys. **85**, 299 (2013)

[6] I. G. Savenko, T. C. H. Liew, and I. A. Shelykh, "Stochastic Gross-Pitaevskii Equation for the Dynamical Thermalization of Bose-Einstein Condensates" *Phys. Rev. Lett* **110**, 127402 (2013).

[7] C. Ciuti, V. Savona, C. Piermarocchi, A. Quattropani, and P. Schwendimann, "Role of the exchange of carriers in elastic exciton–exciton scattering in quantum wells" *Phys. Rev. B* **58**, 7926 (1998).

[8] N. Takemura, S. Trebaol, M. Wouters, M. Portella-Oberli, and B. Deveaud, "Polaritonic Feshbach Resonance" *Nat. Phys.* **10**, 500 (2014).

[9] P. M. Walker, L. Tinkler, B. Royall, D.V. Skryabin, I. Farrer, D. A. Ritchie, M. S. Skolnick and D. N. Krizhanovskii, "Dark solitons in high velocity waveguide polariton fluids", Phys. Rev. Lett. **119**, 097403 (2017).

[10] C. Piermarocchi, F. Tassone, V. Savona, A. Quattropani , and P. Schwendimann, "Nonequilibrium dynamics of free quantum-well excitons in time-resolved photoluminescence" *Phys. Rev. B* **53**, 15834 (1996).

[11] I. Carusotto, and C. Ciuti, "Probing Microcavity Polariton Superfluidity through Resonant Rayleigh Scattering"*Phys. Rev. Lett.* **93**, 166401 (2004).
